# The Medical Life

**Published:** 1902-12

**Authors:** George Munro Smith

**Affiliations:** President, Surgeon to the Bristol Royal Infirmary


"*Scire est nescire, nisi id me*
*Scire alius sciret.*"

DECEMBER, 1902.

### THE MEDICAL LIFE.

The Presidential Address, delivered on October 8th 1902, at the Opening of the Twenty-ninth Session of the Bristol Medico-Chirurgical Society.

BY

GEORGE MUNRO SMITH, M.R.C.S., L.R.C.P., President,

*Surgeon to the Bristol Royal Infirmary.*

ALTHOUGH this Society is a clinical and pathological one, I do not wish now to discuss any medical or surgical problem. With your permission I will leave these subjects alone and give you for a few minutes some thoughts of mine on the daily life of medical men, the joys and sorrows of their calling, and the apparent effects of their work upon their character. I shall only deal with a few points which interest me, hoping that they will interest you; and I shall speak from personal experience, believing that in such matters as these it is more valuable than the most carefully collected experience of others.

We often hear ourselves extravagantly praised or derided: in after-dinner speeches, in testimonials and obituary notices our high-mindedness is insisted on in a manner which is more pleasing than truthful; in fiction, on the other hand, we are generally held up to ridicule in a way we fancy neither pleasing nor truthful. I hope to-night to say what I think, and I wish it to be understood that these few remarks of mine are not intended to serve any special purpose, except to keep you moderately contented for half an hour: nevertheless, it seems to me that it is a good thing sometimes to turn aside from the hurry and impatience of our lives and quietly consider our position; that is what I wish to do to-night.

I suppose the sorrows and joys of the medical life are pretty evenly balanced; it depends here, as always, upon a man's temperament. Black care is sure to sit or walk beside us on many occasions as we go about our daily work; we have vast and awful issues in our hands; and if our conscience is awake, our great responsibilities lie heavily upon our souls, making life a very serious affair indeed. Yet there are many bright gleams constantly illuminating the deeps: the great interest of our work, the fact that we have work set before us that we are bound to do; the confidence placed in us by the greater part of the public; and the humour which abounds everywhere. No one with any sense of the ludicrous can belong to our learned ranks without being sometimes overcome by the absurd things one hears and sees amongst our patients and amongst our colleagues.

And that word "colleague" fitly introduces one of the most noticeable advantages of our profession, namely, our *comradeship*. More than any other set of men, to my thinking, we are ready to help one another, by doing each other's work, by willingly giving advice, by mutual encouragement, and by that strict and wholesome rule of etiquette, or call it rather a moral code, that we shall not slander each other. This last we do not always carry out so strictly as we might; we are only mortal—but we seldom do worse than "damn with faint praise"; and I am sure when patients ask what we think of their pet doctor, we seldom hesitate to say that he is in every way excellent. This is not always truthful, but it is a straining of the truth one can easily forgive. A writer in our enterprising local journal, *The Stethoscope*, expresses himself on this subject as follows:—

"One of the first lessons to be learnt is how to talk to the public about other professional men. The aspirant for eminence must *never abuse them.* He must either overwhelm them with hyperbolic praise, or change the subject suddenly as if there were something that had better not be said; or what perhaps is best of all, in the full torrent of his laudation, bring in, with due tact, some faint hint that Mr. So-and-So would be even more commendable if he had some little smattering of knowledge of his profession."

We are not, however, given to praise each other on our skill, except in such admirable societies as this, where a man reads notes on three successful cases, and his paper is referred to as interesting and instructive, and he is congratulated (with perhaps mild irony) on the fact that his patients *did* recover. But a little praise is to some men a very great reward, especially to those who are beginning their career, and not only gives pleasure but does a great deal of good. We all have a pretty hard fight and are in need of friendly encouragement. What, for instance, can be a more terrible ordeal for an apprehensive man than to do his first big operation at a hospital, surrounded, perhaps, by critical students and watchful surgeons? When this crisis came for me I was in that state when every sign of praise or blame is caught at with frantic eagerness. I do not know how I got through it; but I remember, and always shall, with a deep sense of thankfulness, that as I went home from the Infirmary, one of my seniors, in the kindness of his heart, gave me some words of praise, probably undeserved, which made the world brighter for many days. The feeling of good fellowship, so important to a gregarious animal like man in his daily battle, is fostered by a society like this. The man who keeps aloof from his fellows, never exchanging ideas with them or discussing pathology and treatment, soon degenerates and becomes incapable of keeping up to date. The great wave of modern knowledge sweeps by him and leaves him on his lonely rock, contented and ignorant. Whereas if he attend these meetings he is bound to see and hear much that is interesting; moreover, by intercourse with his fellows his corners are rounded off, his fads laughed away, and his love for clinical and pathological work deepened. And he gradually learns too that his best judges are his fellow-workers, not his patients, and that it is to other medical men that he should look for "all help, all sustainment, all reward."

We are always ready, as I have said, to do each other's work; and seeing another man's patients is not always pleasant, especially if one is at all thin-skinned. One occasionally gets rebuffs. The patient asks with a sigh when Mr. So and So is coming home, and looks very depressed when you have to answer, "Not for two weeks." I once was sent for by a lady doctor to give an anæsthetic in a midwifery case. Almost before I entered the room I was received with a loud and jubilant "Thank God, he's come!" which immediately died away into a kind of despairing howl and the—to me—painful explanation that she thought I was a great namesake of mine. Once or twice patients have confided in me the embarrassing news that they are dying to get rid of their own doctor, but don't know how to do it. Probably my patients have frequently said the same of me. This is very trying, but I think the strangest rebuff I ever heard of was given me when I was attending an elderly nervous lady for a Clifton physician. One morning I called and was kept waiting on the door-step whilst someone peeped through the blinds, and a short colloquy was apparently held; the result being that a servant at length opened the door and cheerfully informed me that Miss H. sent her kind regards, but was "too ill to see me this morning!" This is a very pretty instance of unconscious feminine humour. Patients frequently say very amusing things with the most serious intention. They wish to be kind and say "that the medicine does not seem to have hurt them," as if that was something to be very thankful for. During the absence of a well-known Clifton practitioner a patient called on his substitute and explained that she deeply regretted Mr. C.'s absence, as she was so anxious for him to see her husband. "You know," she said, "Mr. C. attended my first husband in his last illness." I may add that Mr. C. has now returned and the lady is, I notice, in deep mourning.

I suppose it was with no idea of deliberate irony that a busy undertaker wrote to one of our past-presidents, asking if he was offended with him in any way, as he had lately put so little business in his hands. I met this undertaker the other day, and he spoke in high terms of this doctor, so I suppose he has now no complaint against him. It is not only patients and undertakers who say funny things without knowing it. I sent a lady to see a specialist once, and he gave her this advice: "You should take a brisk saline purgative every day before breakfast, and you should spend your mornings on the Downs." And you have probably heard of the eminent man who went from curiosity to a great revival meeting, and sat on a bench in the front of the hall. A Salvation Army officer came round and said: "Oh, are you saved?" to which he answered, rather losing his presence of mind: "Oh, no; I'm a Clifton doctor!" The Salvationist also lost his head, and said: "Oh, I beg your pardon!"

The question of fees is a great worry to some of us. It is a pity we have not a more uniform standard. The public must be very puzzled: but people generally come to the conclusion that if a man charges well, he is a good man; if he charges little, he is worth little. My experience is that patients usually are very good in this respect. Sometimes one comes across stingy and unwilling payers. I once performed tracheotomy on a boy with diphtheria. He was gasping for breath, and cyanosed; but the operation relieved him, and he recovered. Yet when the delighted father paid my bill, he struck out the shillings and paid me in pounds instead of guineas, although he quite realised the value of the operation to his boy. During the "silly season" this year, the *Morning Post*, instead of discussing the sea-serpent, opened a correspondence on the fees of medical men, especially surgeons. Most of the letters were very foolish, but two things struck me. One writer pointed out the great difference between England and Germany in this respect, and said, what no doubt is true, that people go abroad more and more for operations, because they can get the thing done so much more cheaply in Berlin than in London. The other statement I noticed was to the effect that no competent surgeon performed a major operation under 150 guineas; which bears out what I have said as to what the public means by "competency."

Quite recently I had a patient with a tubercular lesion of the wrist. I scraped the part, cleansed it, &c., and gave a bad prognosis. As I understood he was poor, I charged a small fee, and got, not a cheque, but a letter saying that the friends had decided to take him to London. He went, and the wrist was again scraped. The London man charged well, and he had to pay also for a rather expensive nursing-home. I have no particular fault to find with this; but I do object to the next step, which was that the eminent specialist who had operated on him has now, on the return, or rather extension, of the disease, taken him into a hospital as an ordinary patient, and throughout has not communicated with me. We must put down the management of fees as certainly one of the worries of our profession.

The laws of medical etiquette have been for the most part wisely framed, and have, I believe, a good influence on our conduct. But they are often carried out more in the letter than the spirit, and they are a little unjust sometimes to patients. It is made very difficult for them to change their doctor, and some men seem to think they have a prescriptive right to their patients and have taken them on a perpetual lease. I believe great harm is done and much ill-feeling encouraged by these ideas. Let patients go to whom they like: if they leave you, it only shows their bad taste; if they come to you from somebody else, it shows their good taste. In either case, do not trouble!

It is certain that with us the battle is not always to the strong, nor the race to the swift. Success depends on so many things besides knowledge and good intentions. It must be very galling to a well-educated man, whose soul is in his work, to wait year after year for even a small income, whilst men of colossal ignorance are not only making handsome fortunes, but are daily gaining the praise of men. One is almost tempted, when one thinks of this, to agree with the poet:

> "It sounds like voices from the land o spirits
> If anyone obtain that which he merits,
> Or any merit that which he obtains.' [1]

But such a mental attitude is a mistake. In the long run a

[1] Coleridge.

kind of balance is struck, and a man finds his level. And it must be remembered that the public has no means of knowing who are good men and who are frauds except from vague hearsay and the trend of fashion. Some men have a kind of "vogue." Their fame spreads from house to house, and they have their day like all other dogs. How *can* the quack-ridden public tell? They naturally judge by manner, appearance, and by what they call "results." If Miss Jones takes "bile beans for biliousness," and is better in two months, the case is quite plain. "Post hoc ergo propter hoc" is a fallacy that the wisest men fall into. One of the greatest intellects of the eighteenth century was, I suppose, Samuel Johnson, and he pinned his faith to an obscure, unqualified practitioner called Levet, whose fees were "sometimes very small sums, sometimes whatever provisions his patients could afford him." "But," says Boswell, "such was Johnson's predilection for him, and fanciful estimation of his moderate abilities, that I have heard him say he should not be satisfied, though attended by all the College of Physicians, unless he had Mr. Levet with him."

Moreover, many people form their opinions from books they read, and I hope some future president of this Society will deal with that interesting subject, the "doctor in fiction." Thackeray's Dr. Goodenough and Sam Huxter, Allan Woodcourt in *Bleak House*, Dr. Parker Peps and Mr. Pilkins in *Dombey and Son*, George Eliot's Lydgate, Barry's McQueen, to say nothing of Bob Sawyer, Benjamin Allan, and a host of others—what farcical attempts they most of them are! The good ones, as has been pointed out to me, always doing the worst thing for the patient in the kindest possible manner, the rest preternaturally pompous or idiotic; hardly one is at all natural; I do not recognise any of my friends amongst them. Most of the great novelists have very little confidence in us; but one cannot but be struck by the absolute faith that most people place in their doctor. It is one of the blessings, as it is one of the drawbacks, of our profession. If a man goes round with a kind manner and a little address, his patients learn to love him and think him a saint, when in reality he may be utterly unfitted for his work and may be doing great harm.

Faust, you may remember, describes how his father attended people in the pestilence, and was worshipped as a great healer, whereas he killed as many by his treatment as the plague itself did.[1] Some men undoubtedly exercise what may almost be called hypnotism on their patients. I have seen, with astonishment, an operation performed after the mere application of a solution of cocaine to the unbroken skin, absolutely painlessly, "suggestion" apparently causing the anæsthesia, which certainly cocaine, applied in this way, will not do. This confidence must be considered, I think, one of the joys of our profession; it only becomes a sorrow when it is taken from us and given to one of our colleagues.

A great advantage of our calling is its absorbing interest, the interest of intense reality. Disease and Death are the sternest of facts; and in dealing with them we feel we are grappling with forces of Nature, not with theories of laws or morals. In the presence of "the shadow feared by man" everything else is dwarfed into insignificance; and if we look upon our work from a scientific standpoint, there can never be any monotony or ennui. Behind it all, too, is the human element which raises it to a higher level. We are dealing with applied science, but also with the highest product of Nature—the human body.

We are very happily situated in this way, too, that our education and training so well fit us for enjoying other intellectual pleasures; a medical man has excellent facilities for taking up interesting hobbies. To begin with, he has enough general education to appreciate the literature of his own country, and in some cases of other countries. That we have not oftener the taste or inclination to do so is, to my mind, a great pity. We are behind the Church and the Law, I think, in this respect, and thereby lose one of the greatest pleasures of life, and one that is, as I have said, so easily within our reach.

Then, our intercourse with all sorts and conditions of men enables us to see many sides of life, to study character, and therefore should enable us to enjoy one class of literature more. Our smattering of science allows us to understand the scientific

[1] Goethe's *Faust*; First Part.

discoveries and controversies of the day—and these controversies, as we know, are apt to take a wide range into all sorts of social, religious, and educational questions. From our daily work we should learn something of evidence, how to sift the true from the false, and how to gauge character and motives. All this enables us to have mental recreation, and to take a wide and reasonable interest in life.

Again, as we come in contact with all classes in professional and then in friendly intercourse, a medical man is in an excellent position to become a social success, if he has any fancy that way; but from the causes I have mentioned our tastes are too catholic to permit many of us degenerating into mere society men with the narrowed mental horizon which this implies. In hospitality and reasonable social intercourse medicals have many opportunities of indulging, and few men are more acceptable at a dinner-table. Perhaps we do not give up enough of our time to social life: there is so much to read nowadays and so much competition in knowledge, that we cannot "possess our souls" like our forefathers. It is with a feeling of envy and regret that one imagines the literary days when men like Garth and Arbuthnot used to meet Pope at the Kitcat Club. Those times and that type of man have gone never to return; but in a different way the doctor has, from his position, great power in society, a power which should be jealously guarded and restrained. He is consulted about everything, and his opinion looked upon as conclusive. He is the repository very often of terrible secrets: the lurking ghost of insanity, the delinquencies of children, the immorality of parents, the bankrupt condition, fears, despondencies, and family griefs—they are all entrusted to him; and it is to his honour that this trust is seldom violated.

Someone has said that "it would be a very good profession were it not for the patients," and no doubt one is sorely tried by some of the people whom one sees professionally. It is, as a rule, those who have the least the matter with them that test our temper most. They are apt to be exacting, querulous, and unreasonable.

In cases of real illness, however, I, for one, am constantly struck by the courage and unselfishness of men and women. This is very noticeable amongst those who are wrongly called "the lower orders," and a hospital ward has always seemed to me a place full of high moral lessons.

Our work is, perhaps, almost too absorbing: we cannot escape from it; day and night we are more or less haunted by an undertone of human suffering; and we are often worried by the thought that we may not have done our best. Thackeray, in a passage many of you know, says: "It is the father who is stricken. The terrified wife looks on while the physician feels his patient's wrist, smothering her agonies as the children have been called upon to stay their plays and their talk. Over the patient in the fever, the wife expectant, the children unconscious, the doctor stands as if he were Fate, the dispenser of life and death. He *must* let the patient off this time, the woman prays so for the respite! One can fancy how awful the responsibility must be to a conscientious man; how cruel the feeling that he has given the wrong remedy, or that it might have been possible to do better; how harassing the sympathy with survivors if the case is unfortunate; how immense the delight of victory!" [1]

These are some of the joys and sorrows of our daily life; let us consider very briefly what effect this work has upon our character.

To begin with, there can, I suppose, be no doubt that the aim of our profession, to cure or relieve the sick, is, in itself, a noble one.

When Heracles and Peleus and the sons of the Heroes came back from their different sports on the slopes of Pelion and told Cheiron how they had spent their day, "Asclepias came with downcast eyes and whispered how he had watched the snake cast its old skin and grow young again before his eyes, and how he had gone down into a village in the vale and cured a dying man with a herb which he had seen a sick goat eat. And Cheiron smiled, and said: To each Athené and Apollo give some gift, and each is worthy in his place; but to this child they have given an honour beyond all honours, to cure while others kill." [2]

[1] Thackeray's *Pendennis*. [2] *The Heroes*, by Charles Kingsley.

However far we have travelled from that beautiful story, nevertheless we may claim this proud honour as our heritage still. But we must discriminate; we must remember that whatever our motives may be, our efforts and results appeal powerfully to our patients, and the full heart finds relief in thanks and praise. But this tribute, however grateful it may be, must not be taken too seriously. We must not think ourselves noble simply because we are told so. As Dr. Johnson says: "The morality of an action depends on the motive from which we act. If I fling half-a-crown at a beggar with intention to break his head, and he picks it up and buys victuals with it, the physical effect is good, but with respect to me the action is very wrong."[1] If I put a dozen different drugs into a patient, thinking that perhaps one of them may, in some occult manner, do him good, and that any way my visits to him will help me to pay my bills and keep my horse, the physical effect may be good, but the motive is not so very exquisite.

We enter our profession partly because we wish to support ourselves. "If our patients live, they pay their fees; if they die, their heirs pay double." As Iago says: "Either way makes my gain." It is right that we should have a wholesome regard for fees, and we are injuring ourselves and even our patients in the long run if we are too lax upon this point. Nevertheless, we must take this into consideration when we discuss the moral standard of our work. Seeing patients is not necessarily heroic.

A man told me that a certain doctor said he frequently saw thirty or forty patients a day. "Think of the good he must do," said my friend; "he deserves a rich reward." I agree with him; I think he deserves "six months' hard." His reward, no doubt, is a large income and a small conscience, the latter decreasing as the former increases. I do not mean to imply that every one who sees patients at this rate and in the same perfunctory manner is deserving of censure—far from it; some of the best and most self-sacrificing men I know do little else than see patients under pressure of time. But think what the effect must be in most cases on a man's character. Tearing round as fast as two horses can carry him, telling one patient

[1] Boswell's *Life of Johnson.*

that he has a "touch of liver," another that he has "congestion of the kidneys," and another "that it might have turned to typhoid if he had not been called in"—never opening a book or examining a specimen—is this man's mind, do you think, expanding under the process, or is he not more likely to become narrowed into a mere half-reflex visiting machine? The visible effect on his appearance and manners is sufficiently grotesque. He becomes stamped with the trade-mark of his avocation; you can tell at once he is medical. He cannot salute you without betraying himself. He says: "How are we this morning?" and you are almost afraid he will thump your chest and ask you to say "ninety-nine" in the public street. No! I cannot think that, so far as seeing patients is concerned, the daily life of such a man does him any good. He may be considered by some a perfect angel; but there is a "soiled glory and a trailing wing," and his aureole has degenerated into a tall hat.

Then, again, must we not admit that if a man has a leaning towards humbug and insincerity, these qualities are fostered and increased by our daily work? The temptation is so great, and there is no one to interfere. This is too wide a subject for me to enter on to-night; but it may be noted that a great deal of so-called "professional humbug" is legitimate, even advisable or necessary. A judge was very severe, I noticed, on a medical man for saying "he gave the medicine as a placebo." Very many of the drugs we give may be classed under this head, I think. I once acted as *locum tenens* to an esteemed friend who held a public appointment and had a sort of dispensary in which he saw his patients. He explained to me that I should find the useful drugs on the left side of the room, the "placebos" on the right. The right side contained several rows of the usual tinctures, infusions, powders, &c., in stoppered bottles, most of which I could not open. But on the left (the useful drugs) side I could find nothing but some rhubarb pills, some more pills labelled "Very strong," a large bottle of iodide of potassium, and in a drawer a very blunt scalpel, some tow, and two dusty splints.

On the other hand if we take the expressed opinions of medical men at such meetings as these, where we meet in friendly criticism of each other's actions, there are few professions so antagonistic to humbug.

We may call ourselves, I suppose, "men of science." Scientific investigation ought to be one of our chief aims; therefore accuracy of observation should be increased by our work, and this should lead to accuracy of thought. This is a great gain. We get to dislike anything uncertain or obscure; but the scientific spirit is for this very reason not an unmixed blessing. Under the heading of inexact, vague and uncertain are many things which are condemned to our detriment. Many of us practically renounce Art, because in its essence it is often outside the pale of scientific analysis and investigation. "For obscure all great Art is—not with the perplexity of subtle speculation, but with the mystery of vital movement."[1] We find, whether this be the reason or not, a great want of appreciation of artistic productions, whether in painting, poetry, or literature generally, amongst medical men—at least, so it seems to me,—and literature especially is looked upon by many of us as an occasional relaxation rather than as a great force which moulds the character and lives of mankind. This, I believe, is a great mistake, because we must be incomplete without the influences which come from artistic literature, and our style of writing becomes stilted and uninteresting—too much of the catalogue type. "What does it matter," it may be urged, "how you express yourself, so long as you put down your thoughts clearly?" Well, it makes this difference, amongst others, that one style can be read with pleasure, the other can hardly be read at all. We pay very little attention to style, yet we are all, in our reading, more or less influenced by it. One of our past-presidents, Greig Smith, was an artist in almost everything he wrote; and when he gave the address in Surgery at the Association meeting here in 1894, from beginning to end his sentences were examples of how immensely Art adds to the charm of written or spoken words. Yet on looking up the criticisms on this address of his, I have found no definite mention of this carefully sought-for and triumphantly-obtained

[1] *Studies in Literature, 1789—1877*, by Edward Dowden.

result—a result for which he always zealously worked. If this neglect is the result of our training, it must be considered one of its drawbacks.

Some important qualities are fostered by our daily work. We gain, I believe, a reverence for duty. However unpleasant our business, we *have* to do it, and this in time engenders a habit of promptly answering to the stern calls which come to us pretty often "to scorn delights and live laborious days." Courage, presence of mind, readiness, tolerance, and a catholic spirit are fostered by our avocation; so also are self-importance, intellectual conceit, and loss of the faculty of wonder and admiration. We become so accustomed to the greatest marvels of structure and function, that we are apt to become followers of that wretched principle "nil admirari."

Conventional morality, or the reputation of it, we are bound to possess; at all events, those of us in general practice must. With the happy specialist this is not so necessary; indeed, a little wickedness, especially if it be eccentric and amusing, is considered in him a sign of genius, and makes him rather respected as a superior person who need not consider the ordinary humdrum rules of conduct, but is in fact rather above all that.

From of old the medical profession has been the propagator of advanced views, not only in science, but also in social and religious movements. During the stormy period when the principles of evolution were brought so forcibly before the world by Darwin's writings, amidst the howls of execration and denunciation that arose from all quarters, the medical profession recognised the truth of the new doctrines and accepted them. This and many other similar instances prove that there is something in our training which gives a broad mental grasp.

I do not know that we become more charitable or kinder by following the medical profession; it is part of our duty to appear so, and we are judged by appearances. Probably by "assuming a virtue if we have it not," as Hamlet justly says, we gradually become that which we feign, and we therefore gain certain good qualities by the cultivation of their outward signs. As in all other walks of life, the worthiest amongst us are not usually those whose good deeds are most apparent Some of us know what a severe trial of all the Christian virtues it is to see many out-patients. When I was a student I one day witnessed this incident: An out-patient physician had been doing this work for a long time, and was getting into rather an over-wrought mental condition, when a ragged, miserable-looking woman came in with a sick child, who was emaciated and pale. In answer to the injunction to give the child good food, milk and so forth, the woman said something about not affording it, and went sorrowfully away. The doctor made some excuse about seeing her book, and followed her to the door, where, thinking no doubt no one could see him, he gave her some money. The police would strongly condemn this, and perhaps, logically, one cannot defend such an action; but that woman did not think of logic as she went away, and probably the Recording Angel did not.

I set out with two questions in my mind: Is our profession a happy one to follow? and has its daily work a good or bad effect on our intellects and characters? I am inclined to answer the first of these in the affirmative. Whatever hardships we may undergo, I cannot but think that on the whole the joys preponderate over the sorrows. Some of the most obvious pleasures and pains of our work I have purposely not touched upon. The second question I have dealt with too briefly to arrive at any conclusion worth having, perhaps. Personally, I think that as an intellectual training it is rather over-rated by us. As a moral training, it depends on the kind of work a man does, and this is not always a question of choice. Here, again, I can only express my personal belief, which is that a high-class man is much improved by it, a man with sordid aims is made much worse. It is not a profession fit for everyone, and I think it a great mistake for parents to send boys to medical schools who have no liking for the work and are quite unfitted for it. Some talk as if they had a kind of sacred "call" to follow medicine. I should be inclined to say to some of these as the old Scotch mother did to her son who said he had a "call" to the ministry, "Oh, Johnnie, are ye sure it was na some other noise ye heard?"

One other question suggests itself: Is the medical life likely to lead to reasonable worldly success? We in Clifton and Bristol enjoy the great honour of having more doctors to the number of inhabitants than in any other English town; yet in spite of this there is a low death-rate. It is probable that many of us do not make much, financially, out of it. There are no high positions with handsome salaries as in the Church and the Law. But we must not complain; and, after all, the Englishman's idea, that to make money and hold a high position is heaven, or to be poor and have no social position is hell, may not be quite correct. In that address of Greig Smith's which I have spoken of, he says (and what he writes of the surgeon applies also to the physician): "There is no academy to bestow gold medals on the surgical artist, no crowds to gather round to admire his finished work. In the finest and purest sense the surgeon's art is an art of the home, almost of the fireside, it does not appeal to the populace. The surgeon's highest reward is the moistened eye and the tremulous grip of the hand, which bespeak thankfulness for the bread-winner restored to the home, the mother to the children, the child to the parents. The art of the surgeon has its best reward in blessings, not in fame."

The fame acquired in our profession does not as a rule last very long; here and there some great discovery hands down a man's name to posterity, but for the most part even the leaders of our ranks have a very brief immortality; and the majority of medical men, however heroic their work may be, leave no record behind them, even less than those common soldiers who have given their lives for the empire and lie buried in alien ground, watched over by the Southern stars, for *they*, at least, are remembered in the thankfulness of a great nation. But a little reflection tells us that, however humble a position we may hold, our memory is certain to be kept alive in some grateful hearts, and we may all hope to

> "join the choir invisible
> Of those immortal dead, who live again
> In lives made better by their presence." [1]

[1] George Eliot.

